# Arthroscopic Findings of the Joint Distraction for the Patient With Chondrolysis of the Ankle

**DOI:** 10.1155/DTE.4.101

**Published:** 1997

**Authors:** Katsuaki Kanbe, Atsushi Hasegawa, Kenji Takagishi, Hiroyuki Kaneko, Yasuyuki Nakajima

**Affiliations:** 1 Department of Orthopaedic Surgery Gunma University School of Medicine 23-39-22 Showa Gunma Maebashi 371 Japan; 2 Department of Orthopaedic Surgery Gunma Chuo General Hospital 1-7-13 Koun Gunma Maebashi 371 Japan

## Abstract

This case report describes arthroscopic findings of the effect on articular distraction of ankle joint by means of external fixator for the patient with chondrolysis. Arthroscopy showed fibrocartilage tissue lying between the talus and tibia to protect damaged articular surfaces although apparent repair of surface cartilage failed to find.


*Diagnostic and Therapeutic Endoscopy*, Vol. 4, pp. 101–105
Reprints available directly from the publisher
Photocopying permitted by license only

© 1997 OPA (Overseas Publishers Association)
Amsterdam B.V. Published under license
in The Netherlands under the Harwood Academic
Publishers imprint, part of The Gordon and
Breach Publishing Group.
Printed in Singapore.

### Arthroscopic Findings of the Joint Distraction for the Patient with Chondrolysis of the Ankle

KATSUAKI KANBE[a,*], ATSUSHI HASEGAWA[b], KENJI TAKAGISHI[a], HIROYUKI KANEKO[a] and YASUYUKI NAKAJIMA[a]

[a] *Department of Orthopaedic Surgery, Gunma University School of Medicine, 23-39-22 Showa, Maebashi, Gunma, 371 Japan;*
[b] *Department of Orthopaedic Surgery, Gunma Chuo General Hospital, 1-7-13 Koun, Maebashi, Gunma, 371 Japan*

*(Received 7 March 1997; In final form 2 June 1997)*

**This case report describes arthroscopic findings of the effect on articular distraction of ankle joint by means of external fixator for the patient with chondrolysis. Arthroscopy showed fibrocartilage tissue lying between the talus and tibia to protect damaged articular surfaces although apparent repair of surface cartilage failed to find.**

*Keywords:* Arthroscopy, Chondrolysis, Joint distraction, Ankle

### CASE REPORT

A 19-year old woman had the insidious onset of pain in the right ankle joint, and an associated limp was observed. She had no history of previous injury of the right ankle and had no previous injection of steroid. In addition, she had no other known systemic disease or skeletal affliction or previous trauma. Familial history was negative. Date of onset was April 1993, and date of first examination was August 16, 1993. Physical examination revealed swelling and tenderness over the anteromedial aspect of the right ankle. The ankle showed limited range of motion, especially in dorsiflexion, a position that increased her discomfort. There was no crepitus or instability of deltoid and lateral ligament. There was no polyarthralgia and morning stiffness. The laboratory findings were negative of rheumatoid factor and the inflammatory reactions which suspect rheumatic arthritis. Joint fluid was examined for bacterial cultivation and no bacteria was found. The roentgenographic observations were those of capsular distention, slight osteoporosis, and a decrease in cartilage thickness to 0.8 mm on the affected side as compared with 3.2 mm on the normal side (Fig. 1A). With the acute onset of narrowing of the ankle joint space mortise with pain and limited range of motion in such a young patient with no history of trauma and no findings of septic arthritis, we diagnosed chondrolysis of the ankle.

Although we performed conservative treatment with the application of a short-legged walking cast for three weeks, the patient still complained of an

* Corresponding author. Tel.: 81-272-20-7111. Fax: 81-272-20-8275.

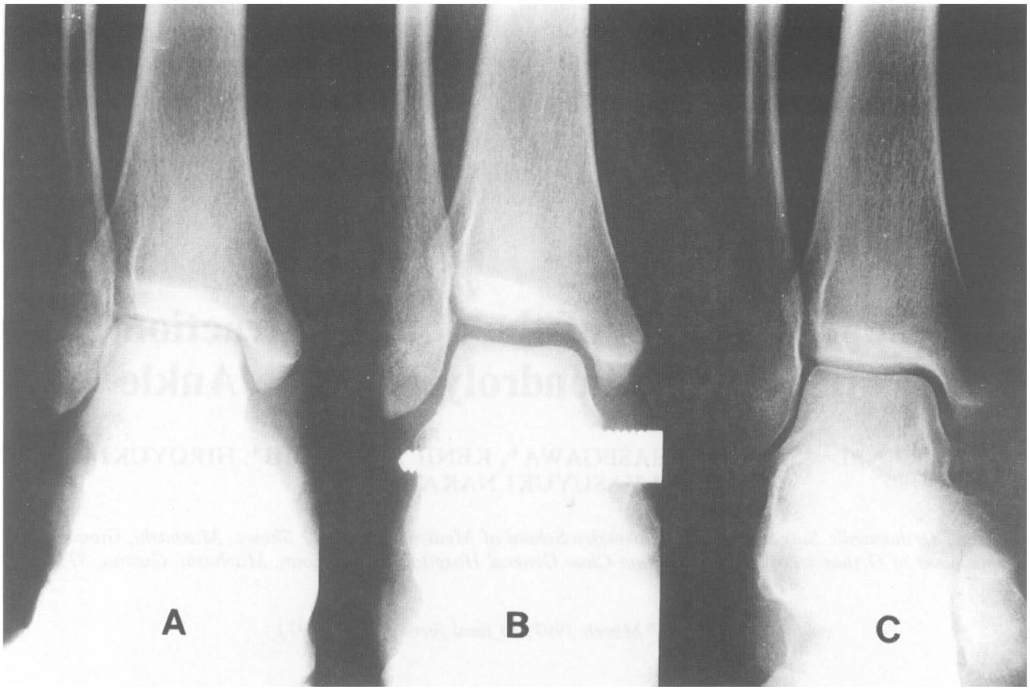

FIGURE 1 A: Preoperatively, the roentgenographic observations were those of slight osteoporosis and a decrease in cartilage thickness to 0.8 mm on the affected side as compared with 3.2 mm on the normal side; B: The joint space of the ankle was distracted to 5.0 mm; C: Twelve months after operation, joint space was 2.5 mm and osteoporosis was not recognized at the subchondral bone.

associated limp and had noticed stiffness of the ankle. We decided to perform arthroscopy of the ankle. The arthroscopic findings were fibrillation and degeneration of cartilage surface over talus and tibia, and partially the erosion of cartilage with synovium proliferation (Fig. 2A). Synovial fluid to perform analyses of immunoglobulins and to submit the fluid for bacterial and viral cultures were performed. No bacterial and viral findings were obtained. Synovium in the joint space was removed and later the pathological report was synovial inflammation. We decided to perform joint distraction of the ankle by external fixation with a lengthener unit in order to attempt to recover sufficient articulation through the reconstruction of the articular surfaces, at the same time as eliminating pain (Fig. 1B). We used Orthofix standard model with ankle articulated body and distraction to 5 mm of the joint space removed 4 weeks after operation. When the fixator was removed, we performed second-look arthroscopy of the ankle with the permission of the patient. On the arthroscopic finding the ankle joint was filled with the formation of fibrocartilage on the damaged articular surfaces and no healed cartilage surfaces were found. The soft tissue lying between talus and tibia was elastic and soft (Fig. 2B). The pathological finding of the lying soft tissue was fibrocartilage with no inflammation (Fig. 3). The roentgenographic observations were the joint space of 2.8 mm after operation and not showing osteoporosis (Fig. 1C). Range of motion was started immediately and partial weight bearing was permitted from the beginning with external fixator. She returned to work 12 weeks after surgery. Follow-up examination 3 years after surgery revealed that the patient had no complaints with reference to the right ankle. The physical examination, including ankle and subtalar motion, was normal.

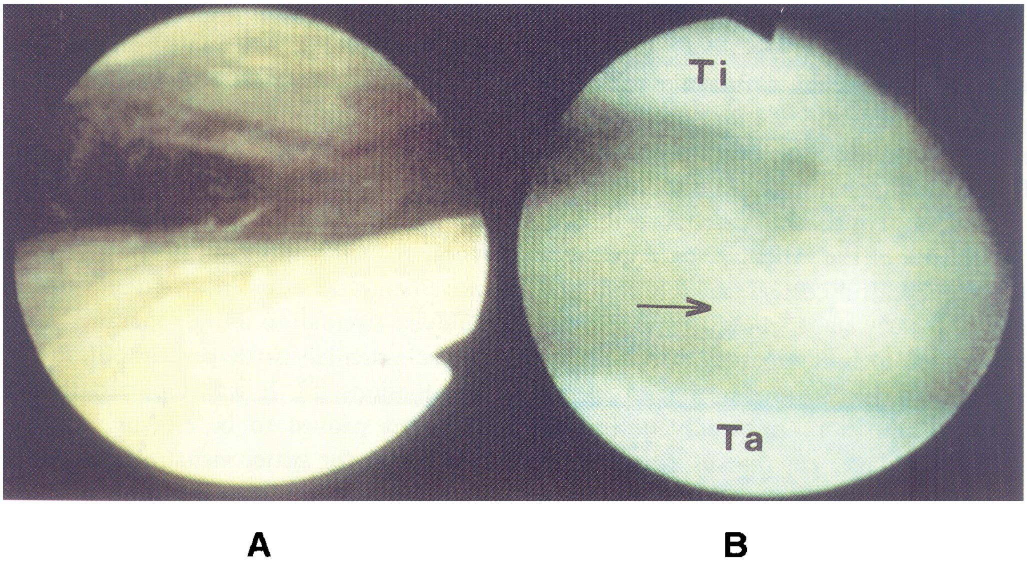

FIGURE 2 A: Arthroscopic findings at the time of the distraction of the ankle joint. The fibrillation of the cartilage surface over talus and tibia, and partially the erosion of cartilage was found; B: At the time of removing the external fixator for second-look arthroscopy, fibrous soft tissue lying between talus and tibia on the damaged articular surfaces (arrow). Ti: tibia, Ta: talus.

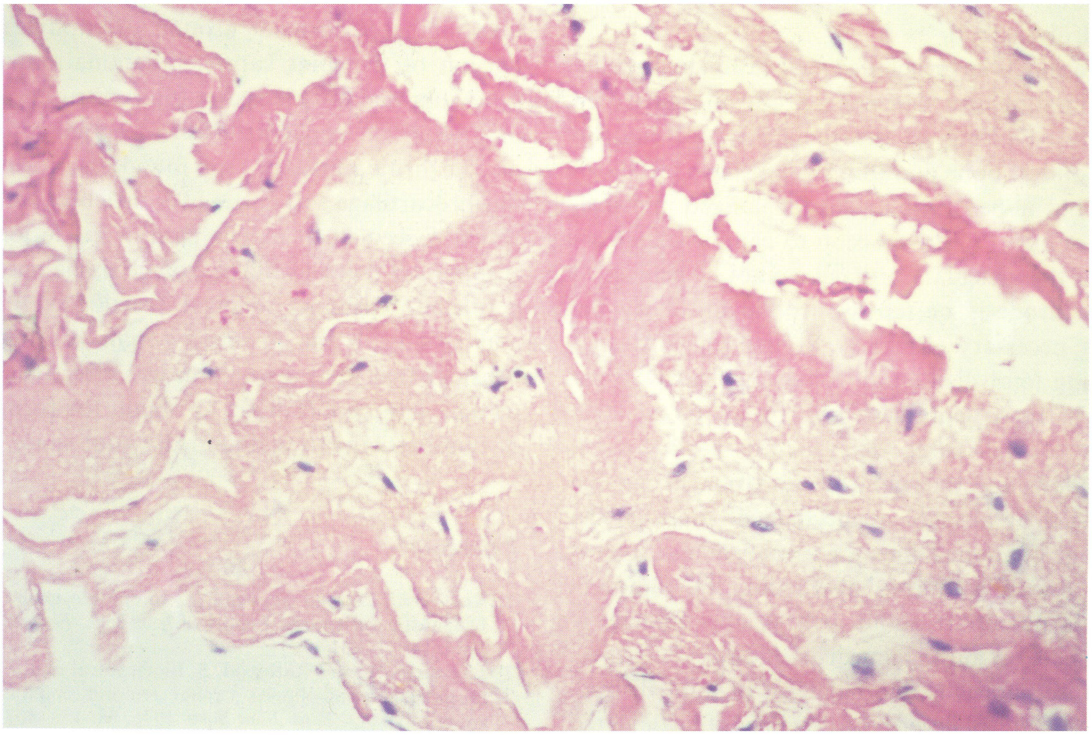

FIGURE 3 Histologic examination of the soft tissue lying between talus and tibia shows fibrous cartilage contained a few fibroblasts (× 200).

### DISCUSSION

Chondrolysis of the articular cartilage of the ankle is an incompletely understood entity. Initial symptoms are pain, swelling, limp, and limitation of range of motion of ankle joint. The radiographic features include a juxtacortical osteoporosis, progressive narrowing of the articular space and erosion of the subchondral cortexes of the tibia and talus. At least in early stage, chondrolysis appears to be more related to inflammatory arthritis than to degenerative arthritis. Surface cartilage, dependent on synovial nutrition, is rapidly destroyed, whereas basal cartilage, capable of receiving at least some nutrition from bone, may be preserved initially. Early attempts at repair in chondrolysis appears to be fibrocartilaginous replacement as contrasted to the clefting and hypercellularity that occurs in degenerative arthritis [1]. Based on this evidence and changes in immunoglobulins, an immunologic etiology and interference with synovial nutrition have been considered the cause for chondrolysis [2,3].

Treatments have included medication to decrease inflammation and pain and exercise under reduced load [4]. Drilling of the remaining cartilage to subchondral bone and joint lavage have also been used, but none of these gives prolonged improvement or cure [5]. Progression to complete destruction of the articular cartilage cannot be prevented and arthrodesis or replacement arthroplasty becomes necessary. Recently it was reported that applying joint distraction using an Ilizarov apparatus in 11 patients with post-traumatic osteoarthritis of the ankle delayed the need for an arthrodesis and that clinical improvement may be produced by absence of mechanical stress on the cartilage combined with the intra-articular hydrostatic pressures during distraction [6], although there is no report about arthroscopic finding of the effects on ankle joint distraction.

Arthrodiatasis is another term of joint distraction coined to describe a regime of articulated distraction and open surgery of the hip employed in Verona since 1981 [7]. Arthrodiatasis is derived from the Greek *arthro* (joint), *dia* (through), and *tasis* (to stretch). Articulated distraction provides off-loading of muscle and body forces and distraction of the joint space by means of an external fixator that crosses the joint [8]. Saggital plane ankle movement is encouraged by the addition of a hinge. Creating a space between the bony surfaces, reducing mechanical stress, and providing movement are initial in an attempt to restore the synovial circulation and encourage fibrous repair of the articular cartilage without the formation of adhesions [8]. It was reported that the ankle distracter proved to be effective in opening the joint space for better visualization, but complications of pin bending, excessive ligament strain, and bony destruction did occur within the clinically recommended range and the safest method of distraction was to use forces $< 135\,N$ in the neutral position [9]. We used the distraction method within clinically safe range for our patient.

The origin of the pain is varied and multiple, including microfractures, stretching of nerve endings in the periosteum around osteophytes, distension of the joint capsle, inflammation of the synovium and muscle spasm [6]. Arthroscopically, in spite of no apparent effect of actual repair of osteoarthritic cartilage, we found the formation of fibrocartilage soft tissue over articular surfaces, which is seemed to reduce pain of ankle joint. It is supposed that this fibrocartilage tissue may play a role of "cushion" to protect articular surfaces and to absorb the force of weight bearing. We consider that joint distraction was effective in our patient with chondrolysis to obtain the formation of fibrocartilage to maintain the joint space.

### *References*

[1] Kozlowski, K. and Scougall, J. Idiopathic chondrolysis: Diagnostic difficulties. Report of four caes. *Pediatr. Radio* 1984; **14**: 314–317.

[2] Eisenstein, A. and Rothscild, S. Biochemical abnormalities in patients with slipped capital femoral epiphysis and chondrolysis. *J. Bone Joint Surg.* 1976; **58**: 459–467.

[3] Yoshioka, Y. and Shichikawa, K. Autoimmunity and chondrolysis of the hip. A report of two cases. *Int. Orthop.* 1987; **11**: 289–293.

[4] Altmann, R.D., Kapila, P., Dean, D.D. *et al.* Future therapeutic trends in osteoarthritis. *Scand. J. Reumatol. Suppl.* 1988; **77**: 37–42.

[5] Videman, T. Connective tissue and immobilization: Key factors in musculoskeletal degeneration? *Clin. Orthop.* 1987; **221**: 26–32.

[6] van Valburg, A.A., van Roermunt, P.M., Lammens, J. *et al.* Can Ilizarov joint distraction delay the need for an arthrodesis of the ankle? *J. Bone Joint Surg.* 1995; **77B**: 720–725.

[7] Aldegheri, R. Arthodiatasi d'anca. *Ortopedia E Traumatologia Oggi* 1981; **1**: 103.

[8] DeBastiani, G., Aldegheri, R., Brivio, L.R. *et al.* The treatment of fractures with a dynamic axial fixator. *J. Bone Joint Surg.* 1884; **66B**: 535.

[9] Albert, J., Reiman, P., Njus, G. *et al.* Ligament strain and ankle joint opening during ankle distraction. *Arthroscopy* 1992; **8**: 469–473.

